# Interactome and F-Actin Interaction Analysis of *Dictyostelium discoideum* Coronin A

**DOI:** 10.3390/ijms21041469

**Published:** 2020-02-21

**Authors:** Tohnyui Ndinyanka Fabrice, Thomas Fiedler, Vera Studer, Adrien Vinet, Francesco Brogna, Alexander Schmidt, Jean Pieters

**Affiliations:** Biozentrum, University of Basel, Klingelbergstrasse 50, 4056 Basel, Switzerland; nf.tohnyui@unibas.ch (T.N.F.); tommyfiedler@gmail.com (T.F.); vera_studer@gmx.ch (V.S.); a.vinet@lady-green.com (A.V.); brogna.francesco@hotmail.com (F.B.); alex.schmidt@unibas.ch (A.S.)

**Keywords:** *Dictyostelium*, coronin A, interactome analysis, Actin

## Abstract

Coronin proteins are evolutionary conserved WD repeat containing proteins that have been proposed to carry out different functions. In *Dictyostelium*, the short coronin isoform, coronin A, has been implicated in cytoskeletal reorganization, chemotaxis, phagocytosis and the initiation of multicellular development. Generally thought of as modulators of F-actin, coronin A and its mammalian homologs have also been shown to mediate cellular processes in an F-actin-independent manner. Therefore, it remains unclear whether or not coronin A carries out its functions through its capacity to interact with F-actin. Moreover, the interacting partners of coronin A are not known. Here, we analyzed the interactome of coronin A as well as its interaction with F-actin within cells and in vitro. Interactome analysis showed the association with a diverse set of interaction partners, including fimbrin, talin and myosin subunits, with only a transient interaction with the minor actin10 isoform, but not the major form of actin, actin8, which was consistent with the absence of a coronin A-actin interaction as analyzed by co-sedimentation from cells and lysates. In vitro, however, purified coronin A co-precipitated with rabbit muscle F-actin in a coiled-coil-dependent manner. Our results suggest that an in vitro interaction of coronin A and rabbit muscle actin may not reflect the cellular interaction state of coronin A with actin, and that coronin A interacts with diverse proteins in a time-dependent manner.

## 1. Introduction

The coronin protein family is comprised of a group of evolutionary conserved proteins that are characterized by the presence of a central Tryptophan-Aspartate (WD or WD-40) repeat-containing domain fused via a linker of variable length to a coiled-coil domain that is involved in homo-oligomerization [1,2]. Coronin molecules are widespread in eukaryotes, with a bioinformatic analysis defining over 723 coronin molecules from 358 different eukaryotic species [3]. Notably, while lower eukaryotes such as yeast, amoeba and parasites including *Leishmania*, *Toxoplasma* and *Plasmodium* appear to express one or maximally two coronin molecules [4,5,6], in higher eukaryotes, multiple coronin molecules are expressed, with up to seven coronins expressed in mammals [1,7,8,9].

The biological function for many of the coronins within cells or organisms remains unclear. While a number of studies have demonstrated an interaction of coronin molecules with actin in vitro, most of the work linking coronin molecules to F-actin interaction has been performed using recombinantly expressed *Saccharomyces cerevisiae* coronin (Crn1) [4,10]. In vitro, Crn1 was found to co-precipitate with F-actin [11], which is in accordance with the presence of a CA-like (Central region fused to an acidic region) domain in yeast Crn1 [12] that is known to be responsible for interactions with actin and Arp2/3 [13,14]. However, the CA-like domain is missing in most other coronin molecules, and in fact, it is unclear to what degree yeast Crn1 is a functional homologue of *Dictyostelium* and mammalian coronins [3]. Furthermore, yeast cells lacking Crn1 do not show an obvious phenotype and have no detectable defects in actin-based processes under a variety of different growth conditions [4,15]. In the unicellular parasites *Toxoplasma gondii*, *Plasmodium* and *Leishmania*, coronins appear to play divergent roles; while in *Leishmania*, coronin regulates microtubule remodeling during cytokinesis [16], in *Toxoplasma gondii*, deletion of coronin does not affect a number of actin-dependent processes, although a weak interaction with actin was observed in vitro [6]. Similarly, *Plasmodium* coronin only weakly interacts with actin in vitro [5] and was shown to localize within the cell in a calcium-dependent and actin-independent manner [17].

In mammals, coronin molecules are emerging as multifunctional regulators of diverse physiological processes, and a common molecular function for the different coronins has not been clearly established. Thus far, F-actin modulation has been the common denominator to explain the role of the different coronin proteins; whereas several coronin proteins were shown to bind F-actin in vitro and within cells [18,19,20,21], other coronin proteins were specifically shown to neither bind to nor modulate F-actin within cells [22,23]. Notably, for one of the best characterized coronins, mammalian coronin 1 (also known as P57 or TACO, for Tryptophan Aspartate containing Coat protein) [24,25], as well as a number of other coronins, emerging evidence is suggesting that they perform actin-independent functions that include neuronal signaling, T cell homeostasis and the initiation of multicellular differentiation [6,26,27,28,29,30].

Given the above-mentioned conflicting reports on the capacity of coronin proteins to interact with F-actin in vitro and within cells as well as the issue of potential redundancy, for example, in mammals, where multiple coronin molecules can be co-expressed [1], we turned to *Dictyostelium discoideum,* that only expresses a single short coronin, coronin A. Coronin A was initially described as a myosin-actin co-precipitating protein that accumulates at crown-shaped, actin-rich cell protrusions (hence the name ‘coronin’) although subsequent work showed that ‘crowns’ are also formed in the absence of coronin A [31,32]. *Dictyostelium* cells lacking coronin A show pleiotropic defects in cytokinesis, uptake of yeast particles as well as motility and migration [33,34,35]. In addition to coronin A, *Dictyostelium* also expresses a ‘tandem’ coronin molecule, termed coronin B, and the two coronins appear to have non-redundant functions [36]. *Dictyostelium* cells, which are a unicellular species when sufficient food is available, have the remarkable capacity, upon starvation, to transform into multicellular structures, resulting in spore-bearing fruiting body formation to ensure long-term survival. The developmental program responsible for the transformation from single cells to spores is initiated upon starvation and depends on cell density and food-deprivation factors that induce pulsating release of cyclic Adenosine Monophosphate (cAMP). This cAMP-release induces the upregulation of genes necessary for cAMP production and chemotaxis, driving the initiation of multicellular development [37,38,39]. Recent work showed that coronin A is responsible for the initiation of the cAMP relay that is required for development upon starvation, but dispensable for cAMP sensing, chemotaxis, and development per se [30]. Together with the finding that F-actin depolymerization does not compromise cAMP-mediated signal transduction, these results suggest that coronin A does not directly modulate F-actin during multicellular development [30]. Instead, F-actin-dependent processes may occur downstream of the coronin A-dependent starvation response, and, in accordance with the role for coronin 1 in mammals and coronin in *Plasmodium*, a prime role for coronin A in *Dictyostelium* may lie within the regulation of cAMP-dependent signal transduction [30]. 

In this paper, we characterized the interactome of coronin A by affinity purification followed by mass spectrometry as well as analyzing the interaction of *Dictyostelium* coronin A with F-actin within cells and with rabbit muscle F-actin in vitro. We found that while the interactome analysis revealed the co-precipitation of coronin A with a number of actin-interacting proteins as well as a transient interaction with the minor actin10 isoform, within cells, coronin A failed to interact with actin under conditions in which actin robustly interacted with myosin. In accordance with *Dictyostelium* coronin A being dispensable for F-actin modulation, phagocytosis of bacteria and inert beads were unaffected by deletion of coronin A. Together, these data suggest that an interaction of coronin A with the actin cytoskeleton occurs indirectly, and that an in vitro association with rabbit muscle actin may not be indicative for the cellular state of coronin A.

## 2. Results

### 2.1. Coronin A-F-Actin Interaction in Vitro and within Cells

Coronin A was originally identified as an actin-myosin interacting protein and has been suggested to play diverse roles in the regulation of a number of actin-dependent processes [40,41,42]. However, more recent work has suggested that the function for coronin A in initiating multicellular development occurs independently of a role in F-actin reorganization [30]. To investigate an interaction of coronin A with actin, a number of experimental approaches were undertaken to determine the interaction partners of coronin A within *Dictyostelium* as well as to assess the capacity of coronin A to interact with F-actin within cells (with endogenous actin) and in vitro (using rabbit muscle F-actin).

**Figure 1:**
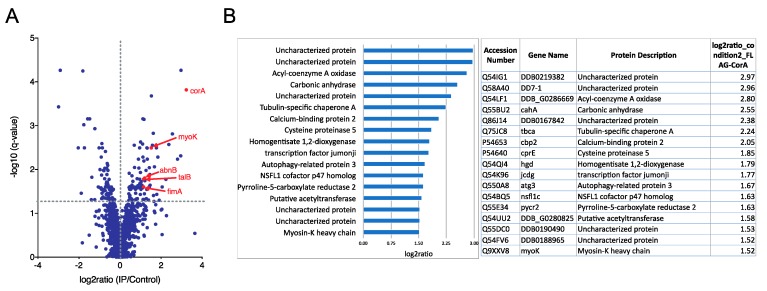
**Coronin A interactome analysis.** Growing *Dictyostelium* cells were collected, lysed and subjected to AP-MS as described in Materials and Methods. Shown are all interacting proteins with log2ratio > 1.5 and *q*-value < 0.05. (**A**). Volcano plot; (**B**). Log2ratiorank. See also Tables S1 and S2.

First, to identify the coronin A interactome in an unbiased manner, *corA*^−^ cells that were transfected with FLAG-tagged coronin A or with non-tagged coronin A (control) and grown in HL5 medium were immunoprecipitated from cell lysates using FLAG affinity chromatography, eluted and interacting proteins analyzed by quantitative mass spectrometry. A total of 47 significantly enriched (log2ratio > 1.5; *q*-value < 0.05) coronin A interacting proteins were identified (Figure 1A,B and Tables S1 and S2). The most prominently associated proteins included several uncharacterized proteins, metabolic enzymes, tubulin chaperones and a transcription factor (Figure 1A,B). While a > 2-fold enrichment for the actin interacting proteins myosin-K heavy chain, actobindin-B/C, talin-B and fimbrin was observed (Figure 1A,B and Tables S1 and S2), actin was not present in the interactome.

**Figure 2:**
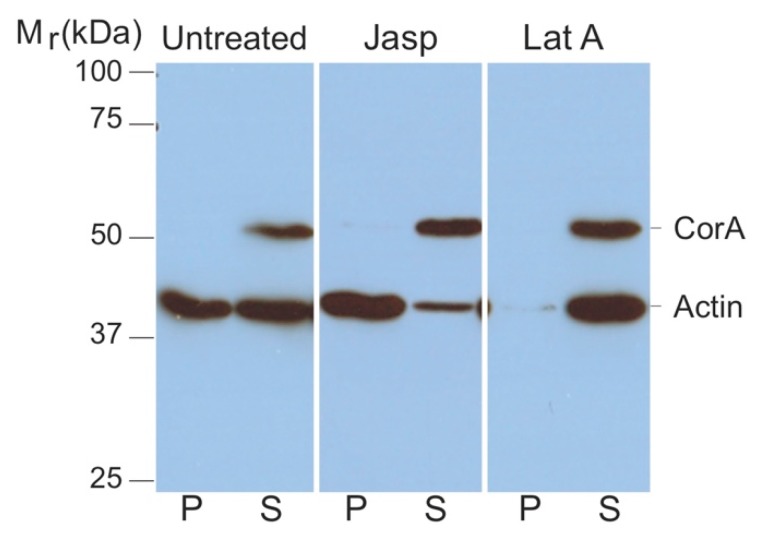
**Coronin A-F-actin interaction within cells.** Cells were either left untreated (**left**) or treated with Jasplakinolide (**middle**) or Latrunculin A (**right**). The cells were lysed in 200 μL F-actin stabilization buffer and F-actin and G-actin were separated by ultracentrifugation of the lysate as described in Materials and Methods. The pellet was resuspended in the exact same volume as the original lysis volume. Proteins in the supernatant (S) or pellet (P) fractions were separated by SDS-PAGE and immunoblotted for coronin A and actin. Shown are representative results from at least four independent experiments.

Since cell lysis prior to mass spectrometry analysis may have disrupted any interactions between coronin A and actin, as a second approach, we analyzed an interaction of coronin A with F-actin within cells. To do so, cells were lysed using F-actin stabilization buffer [43,44,45], followed by analysis of the pellets and supernatants by SDS-PAGE and immunoblotting for actin and coronin A. Since this assay probes the state of F-versus G-actin in situ, care was taken to avoid any dilution factor (see also Materials and Methods). As shown in Figure 2 (‘*untreated*’), all of the coronin A immunoreactivity was recovered in the supernatant, suggesting that at steady state, coronin A does not interact with F-actin. To analyze interaction of coronin A with F-actin under conditions in which F-actin is polymerized, cells were either left untreated or incubated with the F-actin polymerizing drug Jasplakinolide. As a control, F-actin was fully depolymerized by the inclusion of Latrunculin A. Cells were then harvested, lysed, and sedimented followed by analysis of soluble proteins as mentioned above. As can be seen in Figure 2 (*‘Jasp’ and ‘LatA’*), under all conditions of actin polymerization/depolymerization, coronin A was resolved in the supernatant, independent of the polymerization state of actin. 

**Figure 3:**
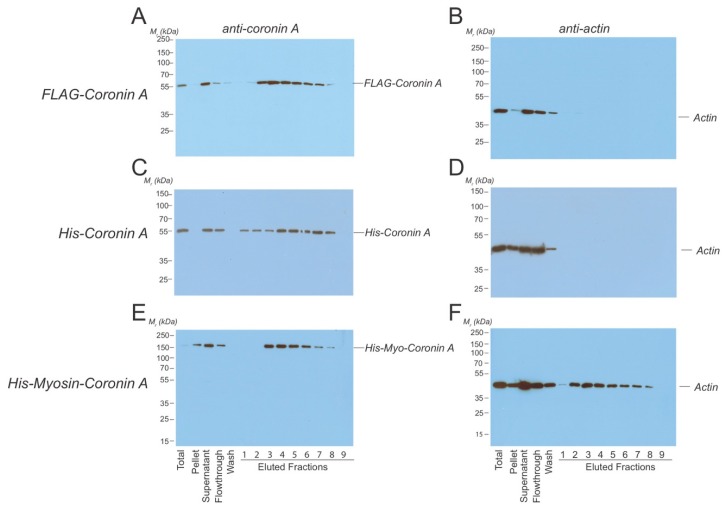
**Co-purification of *Dictyostelium* coronin A and actin.***Dictyostelium* cells expressing the constructs indicated were lysed in lysis buffer and homogenized using a glass Tenbroek homogenizer followed by low speed centrifugation. (**A**,**B**). FLAG-CorA was purified using an anti-FLAG column. Fractions were collected, separated by SDS-PAGE, and tested for the presence of coronin A (**A**) and actin (**B**) by Western blotting. (**C**,**D**). Coronin A fused to a Histidine-tag was purified using Nickel beads. Cells were lysed in the absence of NaCl, fractions were collected, separated by SDS-PAGE, and tested for the presence of coronin A (**C**) and actin (**D**) by Western blotting. (**E**,**F**). Coronin A fused to a Histidine-tagged myosin heavy chain fragment was purified using Nickel beads. Fractions were collected, separated by SDS-PAGE, and tested for the presence of coronin A (**E**) and actin (**F**) by Western blotting. Shown are representative results from at least three independent experiments.

In a third approach, to asses coronin A-F-actin interaction, we directly analyzed whether actin co-eluted with FLAG-coronin A following affinity purification. To that end, the purification procedure as described above for FLAG-coronin A was adapted to ensure that F-actin remained intact by replacing the filtration step (that may have resulted in the clearance of F-actin) by homogenization followed by low-speed centrifugation to remove large debris. Cell lysates were subsequently loaded onto the anti-FLAG column, and following elution with the FLAG peptide, fractions were analyzed by SDS-PAGE and immunoblotted using either coronin A antibodies or anti-actin antibodies. As can be seen in Figure 3A,B, all actin eluted in the flow through, without any actin co-eluting in FLAG-coronin A containing fractions. To test whether the absence of actin in FLAG-coronin A-eluted fractions was due to the FLAG tag, we repeated the purification using His-tagged coronin A (Figure 3C,D). In addition, both the lysis and the elution buffer did not contain any NaCl, given the reported sensitivity of the interaction of *Dictyostelium* coronin A with F-actin to NaCl in vitro [31]. As can be seen in Figure 3C,D, no actin co-eluted with His-coronin A-containing fractions and all the detectable actin signal was found in the flow through. As a positive control, a His-tagged myosin-coronin A fusion protein was expressed in *Dictyostelium*, and this fusion protein was purified by metal affinity chromatography as for His-coronin A. In this case (Figure 3E,F), as expected, actin co-eluted in His-tagged myosin-coronin A containing fractions.

**Figure 4:**
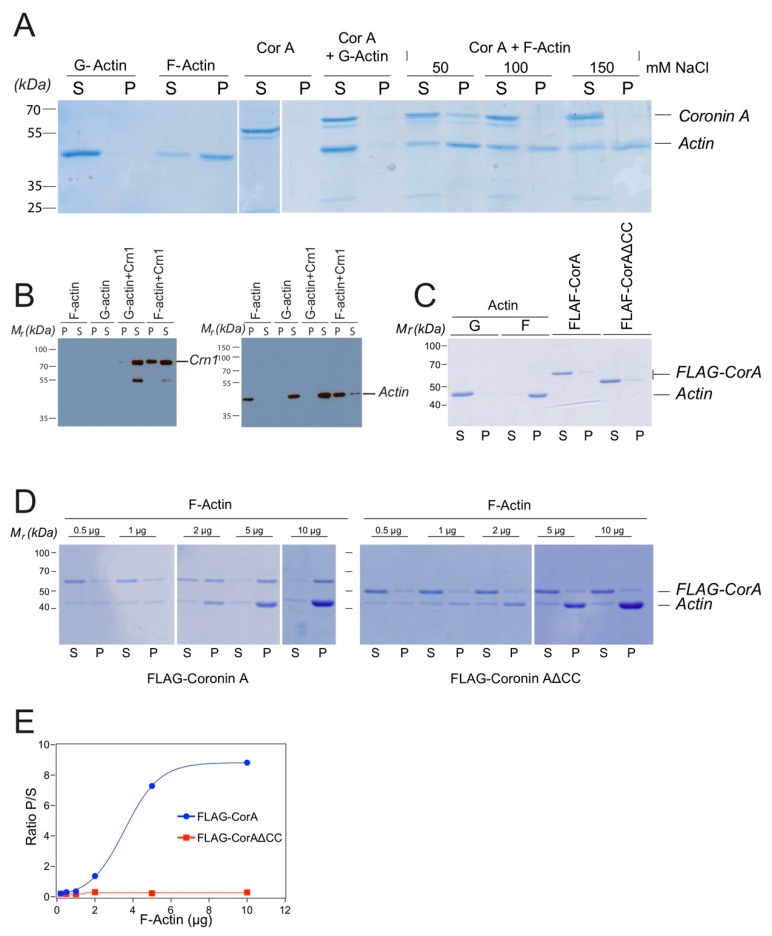
**Coronin A-F-actin interaction with muscle F-actin in vitro.** (**A**). Purified FLAG-CorA and/or equimolar amounts of rabbit muscle G-actin or F-actin was subjected to ultracentrifugation at 100,000× *g*, for 1h at 4 °C in the presence of rabbit muscle G-actin and F-actin and different NaCl concentrations. The supernatant was removed and the pellet resuspended in 2× SDS-PAGE sample buffer. Samples were separated by SDS-PAGE and the gel stained using Coomassie blue. Lanes 1–4: controls; lanes 5–8: coronin A and G-actin; lanes 9–14: Sedimentation analysis was performed in the presence of the NaCl concentrations indicated. (**B**). Rabbit muscle F-actin or G-actin were incubated in the absence or presence of *S. cerevisiae* Crn1, incubated for 20 min at room temperature and the samples were then processed as described in the Materials and Methods. Pellets (P) and supernatants (S) were separated by SDS-PAGE and immunoblotted for Crn1 (**left panel**) or actin (**right panel**) as described above. The lower band most likely represents a degradation product of Crn1. (**C**). Purified actin, FLAG-CorA or FLAG-CorAΔCC were analyzed as in A, separated by SDS-PAGE and the gel stained using Coomassie blue. (**D**). Interaction of the indicated amounts of purified FLAG-CorA (top) or FLAG-CorAΔCC (bottom) with rabbit muscle G- and F-actin was carried out as described in the Materials and Methods. Samples were separated by SDS-PAGE and the gel stained using Coomassie blue. (**E**). Plot of the ratio of rabbit muscle F-actin-bound (co-pelleting, P) to non-bound (S) for FLAG-CorA or FLAG-CorAΔCC determined from the mean grey values of the bands from the Coomassie blue stained gels. Curve fitting shows an apparent Kd of 8.9 μg (CI 5–20 μg). Shown are representative results from at least three (two in the case of panels D, E) independent experiments.

Together, the above data suggest that within cells, coronin A failed to interact with actin; instead, actin may interact with coronin A in an indirect manner, possibly via one or more of the interactors defined by mass spectrometry, such as myosin, fimbrin or talin (Figure 1). However, given the published datasets showing the interaction of coronin A, as well as a number of other coronin molecules with (rabbit muscle) F-actin in vitro [4,5,6,12,31,46,47], we also analyzed the capacity of FLAG-coronin A to interact with purified rabbit muscle F-actin. In addition, given the reported interaction of several coronins with F-actin via their coiled coils [4,18], we included a coronin A mutant lacking the coiled coil domain. We found, in accordance with earlier reports [31], that the interaction of coronin A with rabbit muscle F-actin depended on the ionic strength (Figure 4A), and that coronin A co-pelleted with rabbit muscle F-actin at a concentration of 50 mM NaCl but not at 100 or 150 mM NaCl (Figure 4A). As a control, *S. cerevisiae* Crn1 was employed as it possesses actin interaction domains (in contrast to most other coronins, including *Dictyostelium* coronin A, see [3,12]); as expected, Crn1 readily co-sedimented with rabbit muscle F-actin (Figure 4B). These data suggest that in vitro, coronin A can be co-precipitated with rabbit muscle F-actin under low—but not at elevated ionic—strength conditions.

To further analyze a potential interaction of coronin A with rabbit muscle F-actin, as well as a possible involvement of the C-terminal coiled coil in this process, FLAG-tagged coronin A or coronin A lacking the coiled coil (FLAG-CorAΔCC) was purified and analyzed for co-sedimentation with rabbit muscle F-actin (Figure 4C). As shown in Figure 4D,E, coronin A co-sedimented with rabbit muscle F-actin in vitro in a saturable manner (Figure 4D,E and left panels). Co-sedimentation was dependent on the presence of the coiled coil, since purified FLAG-tagged coronin A lacking the coiled coil did not co-sediment with rabbit muscle F-actin (Figure 4D,E and right panels).

**Figure 5:**
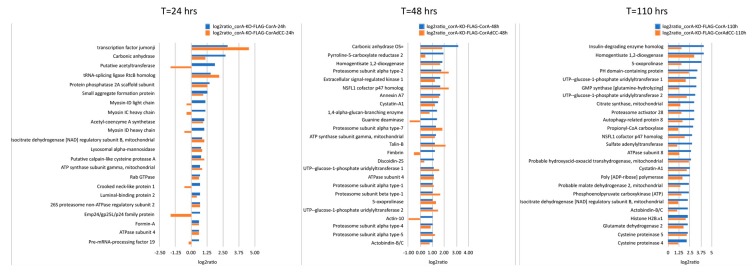
**Time-dependent analysis of the coronin A interactome.** Cells expressing either FLAG-CorA or FLAG-CorAΔCC were sampled at 24, 48 or 110 h, lysed, and subjected to AP-MS as described in Materials and Methods. The 25 most significant interactors (log2ratio > 1; *q*-value < 0.05) are shown. For clarity, proteins with unknown function are not represented. Note that the expression of FLAG-CorAΔCC is ~40-fold enriched relative to FLAG-CorA. See also Table S1.

The here shown in vitro interaction of coronin A with rabbit muscle actin through its coiled coil is in sharp contrast to the absence of an interaction of coronin A with *Dictyostelium* actin (Figure 1, Figure 2 and Figure 3). Therefore, to further investigate a potential interaction of coronin A and *Dictyostelium* actin that may have a temporal aspect and possibly depends on the presence of the coiled coil, we used affinity precipitation followed by mass spectrometry to analyze the interactome of cells expressing either FLAG-tagged coronin A or coronin A lacking the coiled coil (FLAG-CorAΔCC) at the time points shown in Figure 5. Interestingly, there were only a limited number of common interacting proteins among the top 25 hits across the different time points (Figure 5 and Table S1), suggesting a highly dynamic coronin A interactome, at least at this time resolution. Furthermore, we found that for the different time points, a number of actin-interacting molecules were detected in the coronin A interactome, including myosin (24 h), talin, fimbrin (48 h) and actobindin (110 h). In contrast to the in vitro results showing robust interaction with rabbit muscle actin, of the 31 different actin genes expressed in *Dictyostelium* that encode for 15 different isoforms [48,49] we found only the minor actin-10 form to associate with coronin A in a coiled coil-dependent manner at 48 h, but not at 24 or 110 h. We conclude from these data that while *Dictyostelium* coronin A can be co-pelleted in vitro with rabbit muscle F-actin, an interaction of coronin A with actin within cells or cell lysates is not detected; rather, coronin A was found to interact with a range of proteins, including actin-interacting proteins, in a transient manner.

### 2.2. Coronin A is Required for the Phagocytosis of Yeast Particles, but not Bacteria and Inert Beads, Independent of the Coiled-Coil Domain

**Figure 6:**
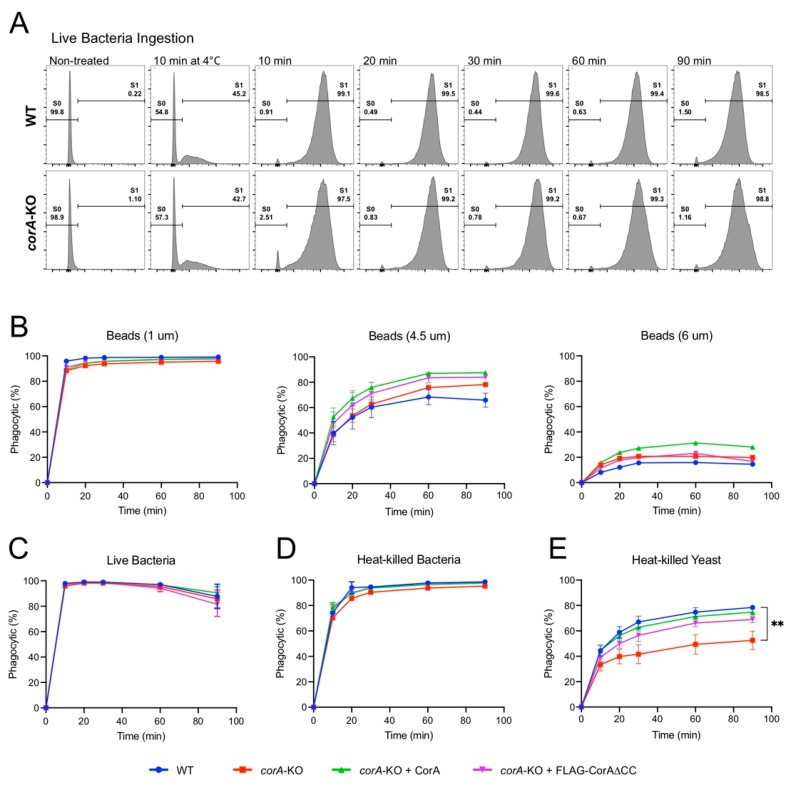
**Phagocytosis in the presence and absence of coronin A.** (**A**): Histogram profiles of the distribution and percentage of wild type and *corA*^−^ cells that have phagocytosed (S1) live *E. coli* after different time points of incubation. Incubation at 4 °C (control) showed significant reduction of phagocytosis. Shown are representative results from at least 3 independent experiments. (**B**–**E**): Plot of the percentage of wild type, *corA*^−^, *corA*^−^ + CorA, *corA*^−^ + FLAG-CorA and *corA*^−^ + FLAG-CorAΔCC DH1-10 cells that have taken up beads of the indicated sizes (**B**) live *E. coli* (**C**), heat-killed bacteria (**D**), or heat-killed yeast (**E**), respectively (error bar = standard error; ** *p* < 0.002).

Together the above data suggest that while in vitro, coronin A can interact with rabbit muscle F-actin in a manner dependent on its coiled coil, it fails to directly bind (F-)actin within cells. To further analyze a potential role for coronin A in F-actin-mediated processes, we assessed the rate of phagocytosis, a process highly dependent on F-actin rearrangement [50,51]. Indeed, blocking actin dynamics using cytochalasin strongly reduced bead uptake, similar to internalization at 4 °C (Figure S1). To determine a role for coronin A in phagocytosis, we assessed the capacity of wild type, *corA*-, or as well as *corA*-cells expressing coronin A or the delta coiled coil mutant to ingest a range of fluorescently labelled particles of different surface compositions and sizes including yeast, bacteria and inert beads using fluorescent activated cell sorting (FACS). We first assessed the ingestion of the natural food of *Dictyostelium* (bacteria) such as live *Escherichia coli* expressing the neon green fluorescent protein (Figure 6A,C) and heat-killed *Klebsiella aerogenes* (Figure 6E), but also of carboxylated inert beads of 1 µm, 4.5 µm, and 6 µm diameter (Figure 6B). No major differences were observed in the phagocytosis of bacteria and inert beads between wild type, coronin A-deficient, or cells expressing full length or delta coiled coil coronin A (Figure 6), suggesting that coronin A as well as the coiled coil is dispensable for phagocytosis of bacteria and inert beads. Interestingly, when internalization of heat-killed *Saccharomyces cerevisiae* was analyzed, cells lacking coronin A displayed significantly reduced levels of phagocytosis compared to wild type cells (Figure 6E), which is consistent with earlier work describing a defect in the uptake of yeast particles in the absence of coronin A [34]. Since yeast uptake in *Dictyostelium* is known to depend on receptor-mediated uptake and is characterized by associated activation of signal transduction [52,53,54], these data are consistent with a role for coronin A in the modulation of signal transduction rather than F-actin rearrangement.

Together, these results suggest that coronin A is dispensable for phagocytosis of bacteria and inert beads, but plays a role in yeast particle uptake in a manner that is independent of F-actin interaction.

## 3. Discussion

Coronins constitute a family of WD repeat containing proteins that are often referred to as F-actin binding and modulating molecules. One reason for this assignment is the fact that coronin A from *Dictyostelium discoideum* was originally identified as a molecule that co-sedimented with an actin/myosin precipitate [31]. However, the evidence for coronin A modulating F-actin within cells is indirect and largely based on the reported phenotypes of *Dictyostelium* lacking coronin A, namely a defect in phagocytosis, chemotaxis and migration [31,32,34,55]. Interestingly, more recent work showed that the defect in chemotaxis and migration in *corA*^−^ cells is readily complemented by pulsing the cells with cAMP [30], suggesting that, per se, these processes do not depend on coronin A. Instead, coronin A was found to be responsible for the initiation of multicellular development [30]. 

Here, we analyzed the interactome of *Dictyostelium* coronin A as well as its interaction with actin. We found that coronin A interacts with diverse proteins in a transient manner and could not find evidence for a direct interaction of coronin A and F-actin within cells or cell lysates. Furthermore, we found a transient association with the minor (<5%) actin10 isoform, but not with actin8, that represents >95% of the cellular actin [48,56]. Since we did detect a number of actin-interacting molecules to co-purify specifically with coronin A, including talin, fimbrin, actobindin and myosin subunits, it is possible that any interaction of coronin A with actin may occur indirectly via these proteins. We also show that the phagocytosis of a range of different cargos was not compromised by the absence of coronin A, with the exception of yeast phagocytosis, which is known to depend on signaling processes [52]. Together these data suggest that coronin A does not directly interacts with/modulates F-actin. 

Most previous studies analyzing coronin–actin interaction have employed rabbit muscle actin to demonstrate in vitro co-pelleting with F-actin. Similarly to earlier studies, we confirmed the in vitro interaction of coronin A with muscle F-actin and further found that this interaction was dependent on the coiled coil, which is consistent with the demonstration that the coiled coil in other coronins also possesses low-affinity actin binding sites [11,57]. Since these latter analyses were also performed using in vitro interaction analyses, it is unclear to what degree this reflects an in vivo association with F-actin; while for a number of mammalian coronins, association with F-actin within cells could not be demonstrated [22,23,45], we cannot, however, also given the high degree of homology between *Dictyostelium* and rabbit muscle actin, exclude the possibility that the presence (within cells) or absence (in vitro) of other factors or differential posttranslational modifications determines coronin A-F-actin interaction. 

The absence of a direct interaction between *Dictyostelium* coronin A and F-actin within cells as shown here is consistent with the finding that coronin A in *Dictyostelium* is dispensable for several F-actin dependent processes: first, as shown here, the phagocytosis of bacteria and inert beads, an exquisite actin-dependent process [50,51], was unaltered in the absence of coronin A. Second, in *Dictyostelium* cells lacking coronin A, F-actin-dependent processes including folate-mediated chemotaxis as well as chemotaxis upon external cAMP pulsing occurred normally [30]. Rather, the reported phenotypes of *corA^–^ Dictyostelium*, including altered chemotaxis, reduced yeast particle phagocytosis, reduced macropinocytosis as well as defective cytokinesis, all of which largely depend on proper signal transduction [54,58,59,60,61], suggests that *Dictyostelium* coronin A may perform a signaling function, consistent with the sequence homology of coronin A with the Gβ subunit of trimeric G proteins as well as the function of mammalian coronin 1 in the modulation of the cAMP protein kinase A pathway [1,26,28,31]. In this light, it is interesting to note that recent work also suggests that the capacity of *Plasmodium* coronin to modulate actin filament turnover occurs in a manner dependent on protein kinase A/cAMP signaling [17].

It is possible that the identified coronin A interacting proteins fimbrin, myosin, talin and actobindin, all of which are known actin interactors [62,63,64,65], function as intermediates to link coronin A to the actin cytoskeleton. For example, the interaction with the F-actin cytoskeleton might serve to regulate a dose- and time-dependent availability and/or potentiation of coronin A for signaling processes such as in multicellular development initiated upon starvation [30,66,67]. In such a scenario, an indirect and labile interaction of coronin A with the cytoskeleton would be advantageous, making it quickly available for incorporation into other complexes.

## 4. Materials and Methods

### 4.1. Cells, Antibodies, and Growth Conditions

DH1-10 wild-type *Dictyostelium discoideum* cells were acquired from dictybase.org. The *corA*-deficient (*corA*^−^) cells are described elsewhere [30]. Cells were grown in HL-5 media [68] in 100 mL or 500 mL Erlenmeyer flasks at 22 °C with 160 rpm. Anti-coronin A antiserum was described earlier [30]_._ Anti-yeast Crn1 antiserum was produced in rabbits using recombinant Crn1 (Thermo Fisher). Mouse anti-actin clone 4 was purchased from Millipore. For production of N-terminal FLAG-tagged (DYKDDDDK) coronin A, full length coronin A-encoding cDNA was amplified from DH1-10 genomic DNA by PCR, using primers to insert a thrombin cleavage site to the 5′ end, and BamHI restriction sites to either end of the PCR product. The thrombin cleavage site was optimized for *Dictyostelium* codon usage. Forward primer: 5′ ATTGGATCCTTAGTTCCAAGAGGTTCAATGTCTAAAGTAGTCCGTAGTAG ‘3; Reverse primer: 5′ ATTGGATCCTTAGTTGGTGAGTTCTTTGATTTTGGGATCCTTTTTAACG ‘3. The PCR product was first subcloned into pCR-BluntII-TOPO vector (Invitrogen), sequenced, digested with BamHI and inserted into the vector pTX-FLAG (dictybase.org). The resulting vector carried a FLAG-tag 10 amino acids upstream of the inserted thrombin cleavage site and coronin A. The expression of the fusion protein is driven by a *Dictyostelium*-actin 15 promoter. For the construction of the coronin A expression vector, the actin15 promoter was synthesized with XbaI restriction sites at both ends and cloned into pUC57 vector (Eurofin genomics); then subcloned into the XbaI site of pBIG (dictybase.org) and checked for correct orientation by sequencing. Then full length coronin A CDS was amplified from DH1-10 genomic DNA by PCR, using primers to introduce BamHI restriction sites on both ends of the PCR product, which was then cloned into the pBIG vector to give pBIG-CorA driven by the actin15 promoter. For ΔCC-CorA, the expression plasmid was generated by synthesizing (Eurofins genomics) BamHI flanked coronin A CDS lacking the last 34 codons, which encode the coiled coil motif, and cloning into the BamHI site of pTX-FLAG vector to generate pTX-FLAG-CorAΔCC.

For the generation of a histidine-tagged (6×) version of coronin A, the coronin A coding region was amplified from DH1-10 genomic DNA with forward (FwCorA HpaI AGAGCGTTAACATGTCTAAAGTAGTCCG) and reverse (RevCorA HpaI AGAGCGTTAACTTAGTTGGTGAGTTCTTTG) primers adding HpaI restriction sites on both ends of the gene. The resulting fragment was ligated into the cloning vector T-easy (Promega) according to the manufacturer’s protocol. The vector for production of N-terminal His-tagged (6× His) coronin A was then generated by removing the GFP sequence from the vector pTX-GFP (dictybase.org) with EcoRV and inserting the full length HpaI restricted coronin A sequence in its place via blunt end ligation. For use as a positive control in the actin co-purification experiments, we generated a vector that expresses coronin A as a fusion protein fused to the C terminus of a 6x His-tagged portion of myosin heavy chain capable of binding actin. pDIC2, the vector containing the myosin heavy chain fragment, was a kind gift from Thomas Reubold of the Institute for Biophysical Chemistry at Hannover Medical School [69,70]. The coronin A-encoding gene was excised from the T-easy cloning vector described earlier using the restriction enzyme HpaI to create blunt ends, pDIC2 was linearized with the blunt-cutting restriction enzyme EcoICRI and the coronin A-coding sequence was ligated via blunt end ligation. For the generation of a yeast Crn1 expression vector, Glutathione S-transferase (GST)-fused Crn1 was amplified from an existing vector (pGAT_Crn1); forward primer: 5′GTGTCTGCAGATGTCCCCTATACTAGGTTATTG’3 and reverse primer: 5′CACACTGCAGTCATTTTGACAGTTCGCC’3. GST-Crn1 was then cloned into a p425-TEF yeast expression vector via PstI sites [71].

### 4.2. Anti-Flag Immunoprecipitation and Mass Spectrometry

Cells (5 × 10^6^, the *corA*^−^ transformed with and stably expressing FLAG-CorA or FLAG-CorAΔCC and *corA*^−^ expressing CorA as control for unspecific binding) were seeded in triplicates at 10^5 cells/mL, grown in HL-5 media as mentioned above and harvested at 24, 48 and 110 h, washed 2 times with ice cold PBS and lysed with 500 µL Lysis Buffer (low salt TBS (20 mM Tris-HCl pH 8.0, 25 mM NaCl, 5 mM KCl)/1% Triton ×100/HALT from Thermo #1861281) for 30 min on ice with gentle agitation every 5 min and clarified for 15 min at 18,200× *g* at 4 °C. Monoclonal anti-FLAG-M2 slurry (Sigma, F1804-50UG, 25 μL) was washed twice with low salt TBS and co-incubated with 450 μL of cleared lysate for 90 min at 4 °C in 2 mL microfuge tubes with 360° rotation. Unbound/non-interacting proteins were removed by 3 washes with low salt TBS. Peptide elution occurred in series, first with 100 μL elution buffer 1 (1.6 M urea, 100 mM Ammoniumbicarbonate, 5 μg/mL trypsin) at 37 °C with 1200 rpm for 30 min and then twice, each with 40 μL elution buffer 2 (1.6 M urea, 100 mM Ammoniumbicarbonate, 1 mM TCEP), vortexing and centrifuging, collecting and pooling the supernatant. Eluted proteins were reduced by adding 9 μL TCEP from a 200 mM stock solution to the pooled supernatant (total volume 180 μL) and alkylated with 3.8 μL chloroacetamide (750 mM stock solution) for 1 h at 37 °C, then digested overnight with 0.5 μg of trypsin (Promega USA). Samples were acidified with 150 μL of 5% TFA, pH < 2. Peptides were bound to acetonitrile conditioned C18-columns, washed with 0.1% TFA and eluted with C18-buffer (50% acetonitrile/50% water (*v*/*v*) and 0.1% TFA). Eluted peptides were concentrated under vacuum to dryness, then dissolved in and adjusted to 0.2 µg/µL with 0.1% formic acid.

For the LC-MS/MS analysis, the μRPLC-MS system was setup as described previously [72]. Chromatographic separation of peptides was carried out using an EASY nano-LC 1000 system (Thermo Fisher Scientific), equipped with a heated RP-HPLC column (75 μM × 37 cm) packed in-house with 1.9 μM C18 resin (Reprosil-AQ Pur, Dr. Maisch). Aliquots of 1 μg total peptides were analyzed per LC-MS/MS run using a linear gradient ranging from 95% solvent A (0.15% formic acid, 2% acetonitrile) and 5% solvent B (98% acetonitrile, 2% water, 0.15% formic acid) to 30% solvent B over 120 min at a flow rate of 200 nl/min. A mass spectrometry analysis was performed on Q-Exactive HF mass spectrometer equipped with a nano electrospray ion source (both Thermo Fisher Scientific). Each MS1 scan was followed by high-collision-dissociation (HCD) of the 20 most abundant precursor ions with dynamic exclusion for 30 s. The total cycle time was approximately 1–2 s For MS1, 3e6 ions were accumulated in the Orbitrap cell over a maximum time of 100 ms and scanned at a resolution of 120,000 FWHM (at 200 m/z). MS2 scans were acquired at a target setting of 1e5 ions, accumulation time of 50 ms and a resolution of 15,000 FWHM (at 200 m/z). Singly charged ions and ions with unassigned charge state were excluded from triggering MS2 events. The normalized collision energy was set to 28%, the mass isolation window was set to 1.4 m/z and one microscan was acquired for each spectrum.

To determine bait-binding affinities, an MS1 based label-free quantification was carried out. Therefore, the generated raw files were imported into the Progenesis QI for proteomics software (Nonlinear Dynamics, Version 2.0) and analyzed using the default parameter settings. MS/MS-data were exported directly from Progenesis QI for proteomics in mgf format and searched against a decoy database of the forward and reverse sequences of the SwissProt entries of *Dictyostelium discoideum* (www.ebi.ac.uk, release date 2017/10/09) and commonly observed contaminants (in total 26,272 sequences) using MASCOT (Matrix Science, Version 2.4.1). The search criteria were set as follows: full tryptic specificity was required (cleavage after lysine or arginine residues); 3 missed cleavages were allowed; carbamidomethylation (C) was set as fixed modification; oxidation (M) as variable modification. The mass tolerance was set to 10 ppm for precursor ions and 0.02 Da for fragment ions. Results from the database search were imported into Progenesis QI for proteomics and the final peptide measurement list containing the peak areas of all identified peptides, respectively, was exported. This list was further processed and statically analyzed using our in-house developed SafeQuant R script (SafeQuant, https://github.com/eahrne/SafeQuant, [72]). The peptide and protein false discovery rate (FDR) was set to 1% using the number of reverse hits in the dataset. All quantitative analyses were performed in biological triplicates. The resulting details of the proteomics experiments carried out including identification scores, number of peptides quantified, normalized (by sum of all peak intensities) peak intensities, log2 ratios, coefficients of variations and *p*-values for each quantified protein and sample are displayed in Supplementary Table S1. All raw data and results associated with the manuscript will be deposited into the Proteome X change Consortium via the PRIDE [73] partner repository with the dataset identifier PXD009483 and 10.6019/PXD009483. 

For the data shown in Figure 1b (Table S2), the log2ratio for FLAG-CorA samples (pooled of all samples from the different timepoints) was determined against the control sample expressing non-FLAG tagged CorA and background proteins were filtered out using the cutoff of log2ratio > 1.5 and *q*-value < 0.05; this dataset reflects the growth phase-independent interactome of coronin A. The significantly enriched proteins ranked per their fold enrichment were plotted using Numbers. For the time-dependent analysis in Figure 5, the log2ratio for FLAG-CorA and FLAG-CorAΔCC against the control sample expressing non-FLAG tagged CorA were calculated and the same cutoff set as above, and the samples for each time point (representing the early log, log and early stationary growth phases, i.e., common to exponential growth and stationary phases) were compared to the control of the same time point. The top 25 significantly enriched proteins ranked per their fold enrichment in FLAG-CorA were plotted using Numbers.

### 4.3. Analysis of F-Actin and G-Actin from Cell Lysates

Coronin A-actin interaction from cell lysates was essentially performed as described [45]. In brief, DH1-10 wild type cells were harvested (4 × 10^6^ cells/sample), washed and resuspended in 0.5 mL starvation buffer B (5 mM Na_2_HPO_4_, 5 mM KH_2_PO_4_, 2.5 mM MgSO_4_, 200 μM CaCl_2_ [74]. The cells were then exposed to either 9 μM Jasplakinolide (Sigma-Aldrich) for 1 h to induce actin polymerization, 10 μM Latrunculin A (Sigma-Aldrich) for 30 min to induce actin depolymerization, or buffer alone as a control and placed on a shaking platform at 22 °C. The cells were pelleted and washed twice with phosphate-buffered saline (PBS), and then lysed with 200 μL F-actin stabilization buffer (50 mM PIPES pH 7.0, 50 mM NaCl, 5 mM MgCl_2_, 5 mM ethylene glycol-bis(β-aminoethyl ether)-N,N,N′,N′-tetraacetic acid (EGTA), 5% glycerol, 0.1% Triton X-100, 0.1% Tween 20, 0.1% NonidetP-40, 0.1% β-Mercaptoethanol, 1 mM ATP, protease inhibitor mix [44,75]) on ice for 15 min. The lysates were pre-cleared by centrifugation at 600× *g* for 5 min and the supernatant was subjected to ultracentrifugation at 150,000× *g* for 30 min at 4 °C to sediment F-actin. The supernatant was removed and the remaining pellet was resuspended in 100 μL ice cold distilled water containing 10 μM Cytochalasin D (Sigma, St. Louis, MO, USA) for 30 min on ice and occasionally agitated gently by pipetting up and down. The resuspended pellet fraction was then mixed with 100 μL 2× F-actin stabilization buffer to bring the solution to the same volume as the previously removed supernatant fraction. Ten microliter of each supernatant (G-actin) and pellet (F-actin) were analyzed by SDS-PAGE and immunoblotting using anti-actin and anti-coronin A antibodies as described below. 

### 4.4. Protein Purifications

For co-precipitation analysis, FLAG-coronin A and FLAG-CorAΔCC were purified using the M2 Flag affinity gel. In brief, 5 × 10^8^ FLAG-CorA- or FLAG-CorAΔCC-expressing cells were harvested in log growth phase and washed twice with ice cooled TBS (20 mM Tris-HCl pH 8.0, 150 mM NaCl, 5 mM KCl). The cells were then lysed in 4 mL lysis buffer (TBS, 2 mM EDTA, 1% Triton ×100, Protease/Phosphatase Inhibitor from Thermo-Fischer #1861281) on ice for 30 min with gentle agitation every 5 min. The lysate was cleared at 18,000× *g* for 15 min at 4 °C, filtered through a 0.45 μM filter (Sartorius), then loaded onto 250 µL of M2-anti-FLAG slurry and incubated with rotation in 15 mL Falcon for 90 min at 4 °C. Non-bound protein were washed off 4× (each with 1 mL lysis buffer), and then 8× with 1 mL each of low salt TBS (20 mM Tris-HCl pH 8.0, 25 mM NaCl, 5 mM KCl) at 1000× *g*, 5 min at 4 °C, while collecting the supernatant and determining the presence of protein at OD280 with an Eppendorf BioSpectrometer^®^ basic (Eppendorf, Hamburg, Germany). By the 9th wash, no protein was detected in the supernatant. Bound proteins were eluted with 250 µL of 3× FLAG peptide (Sigma or GenScript) at 0.2 µg/µL in low salt TBS for 1 h at 4 °C. Purified fractions were run on 10% SDS-PAGE gel, stained with Coomassie^®^ G-250 SimplyBlue™ SafeStain (ThermoFisher) and imaged by scanning with a CanoScan 9000F Mark II scanner (Canon).

G-actin was isolated from rabbit muscle acetone powder (Sigma M6890) as described previously [76]. In brief, 1.5g acetone powder was dissolved in 30 mL buffer G (2 mM imidazole, 0.2 mM ATP, 0.5 mM dithiothreitol (DTT), 0.2 mM MgCl_2_, pH 7.2–7.4) and stirred on ice for 30 min. The extract was filtered and the remaining acetone powder was extracted with another 30 mL of buffer G for 30 min. Both supernatants were pooled and centrifuged for 20 min at 25,000× *g* at 4 °C. The supernatant was pooled and supplemented with KCl (1M stock) to a final concentration of 50 mM and MgCl_2_ to a final concentration of 2 mM and stirred at 4 °C for 1 h. After stirring we slowly added ground KCl powder to reach 0.8 M final concentration and stirred for another 30 min at 4 °C. The solution containing polymerized actin was centrifuged for 1 h at 150,000× *g* at 4 °C and the resulting pellet was resuspended in 4 mL of buffer A (2.5 mM imidazole, 0.2 mM ATP, 0.2 mM CaCl_2_, 0.005% NaN_3_, 0.2 mM DTT, pH 7.2–7.4) using 18G and 23G syringe needles. The dissolved pellets were dialyzed for 3 days against daily exchanged buffer A, followed by ultracentrifugation at 250,000× *g* at 4 °C for 1.5 h. The depolymerized actin in the supernatant was further purified using gel filtration (Superdex 200 10/300 GL, GE Healthcare). The purified G-actin was kept dialyzing against buffer A at 4 °C for two weeks.

For the purification of yeast Crn1, the cDNA coding for Crn1 was fused with that of GST as described above. An overnight culture of Y36032_GST-Crn1 yeast (Euroscarf) was diluted in synthetic dropout complete-LEU medium to an OD600 of 0.2. The cells were grown at 30 °C to an OD600 of 0.8–1 in 3l Erlenmeyer flasks on a shaking platform. The cells were harvested at 5000× *g* for 5 min at 4 °C and washed with ddH_2_O. Pellets from a volume of 100 mL original culture were resuspended in 500 µL yeast lysis buffer (20 mM Tris-HCl pH 8.0, 150 mM NaCl, 2 mM EDTA, 0.1% TX-100, 1 mM PMSF, 3 mM DTT, complete protease inhibitor from Roche) and mixed with 500 µL glass beads (Carl Roth: A553.1 Glasperlen 0.25–0.5 mm). The yeast cells were disrupted in a bead vortex for 2 × 30 s. In between vortexing steps, the lysate was placed on ice for 5 min. The supernatant was removed and the remaining material was extracted a second time with 500 µL yeast lysis buffer. Pooled lysates were centrifuged at 17,000× *g* for 1 min at 4 °C and GST-Crn1 purified on a GSTrap (GE Life Sciences) column followed by incubation with thrombin (4 hrs, 4 °C; 1 unit per 100 µg of protein) to remove the GST-tag. Thrombin was removed using benzamidine beads (50% slurry; Sigma-Aldrich #A7155) and the Crn1 solution was dialyzed (Spectrapor, 10 kDa cutoff) against KMEI buffer (see below) followed by removal of the GST tag using GSTrap from GE and concentrated in an Amicon MWCO 50 kDa device (Millipore, Burlington, MA, USA).

For the co-purification of His-coronin A and His-Myosin-coronin A with actin, the purification was performed as described above with the following changes: cells were lysed in 5 mL imidazole lysis buffer (50 mM Tris-HCl pH 7.5, 20 mM Imidazole, 2 mM Benzamidine, 1 mM EDTA, 0.5% Triton × 100, protease inhibitor mix) on ice for 20 min. The lysate was loaded onto a 300 μL bed of Ni-NTA resin (Qiagen, Hilden, Germany). The loaded column was washed with 40 column volumes of imidazole washing buffer (50 mM Tris-HCl pH 7.5, 40 mM Imidazole, 1 mM EDTA, 2 mM Benzamidine), and bound proteins were eluted with imidazole elution buffer (washing buffer + 260 mM imidazole). Collected fractions were then analyzed by Western blotting as described below.

### 4.5. Coronin A-F-Actin co-Precipitation Analysis 

Coronin A-F-actin co-precipitation analysis was carried out using the FLAG-coronin A or FLAG-CorAΔCC and the procedure according to the Hypermol Actin toolkit (Hypermol, Bielefeld, Germany). In brief, FLAG-CorA and FLAG-CorAΔCC were purified as described above using M2-anti-FLAG resin and dissolved in low salt TBS (20 mM Tris-HCl pH 8.0, 25 mM NaCl, 5 mM KCl). For preparation of rabbit muscle actin, lyophilized G-Actin was reconstituted with 900 μL H_2_O to obtain a 1.1 mg/mL stock solution, and left to rehydrate at room temperature for 5 min, and subsequently dialyzed overnight in MonoMix buffer (0.1 mM CaCl_2_, 0.5 mM DTT, 0.4 mM ATP, 2 mM Tris-HCl, pH 8.2). For F-Actin preparation, the G-actin stock was pre-spun at 100,000× *g*, 1 h, 4 °C in an Optima TLX ultracentrifuge (TLA55) and the supernatant was used for the co-sedimentation assay. G-actin was mixed in a 1:10 ratio with 10× PolyMix buffer (Hypermol, 1 M KCl, 0.02 M MgCl_2_, 0.01 M ATP, 0.1 M imidazole, pH 7.4) and left 30 min at room temperature for polymerization. For co-sedimentation, 2 µg prespun (100,000× *g*, 1 h at 4 °C in an Optima TLX ultracentrifuge (TLA55)) FLAG-CorA or FLAG-CorAΔCC was incubated with different amounts of F-actin for 45 min at room temperature. For higher salt concentrations, FLAG-CorA and G/F-Actin were mixed at equal molar ratios of 1 μM and additional NaCl (from a 5M stock solution in ddH_2_O) was added to the samples to final concentrations of 50 mM, 100 mM and 150 mM in a total volume of 40 μL. After incubation, samples were centrifuged at 100,000× *g*, 1 h, 4 °C in an Optima TLX ultracentrifuge (TLA55, Beckman Coulter). As controls G-actin, F-actin, FLAG-CorA, and FLAG-CorAΔCC were centrifuged separately to assess their solubility. After centrifugation, the supernatants were transferred to 1.5 mL microfuge tubes containing SDS sample buffer (Tris-HCl pH 6.8, 2% SDS, 5% Glycerol, 0.015% DTT, 0.0002% bromophenol blue). The pellet was washed twice with low salt TBS (25 mM NaCl, 20 mM Tris-HCl, pH 7.5) and resuspended in low salt TBS containing SDS sample buffer and transferred into 1.5 mL microfuge tubes. Samples were boiled for 5 min at 95 °C and separated on a 10% SDS-PAGE. The gel was stained using Coomassie G250 SimplyBlue™ SafeStain (ThermoFisher, Waltham, MA, USA). For the sedimentation analysis of yeast Crn1, a stock of freshly purified G-actin was diluted to 4 μM into buffer A. KMEI (10×) actin polymerization buffer was added to yield a 1× concentration (KMEI; 20 mM Imidazole, 50 mM KCl, 1 mM EGTA, 1 mM MgCl_2_, pH 7.5) and the actin was left to polymerize at RT for 1 h. Thirty μL of F-actin or G-actin were mixed with 10 μL yeast-Crn1 purified as described [4] to a final volume of 40 μL and a final concentration of 500 nM. The mixture was incubated for 30 min at room temperature while shaking. The samples were then subjected to ultracentrifugation at 150,000× *g* for 30 min at 4 °C. After removal of the supernatant the pellets were resuspended in 40 μL distilled water with 10 μM cytochalasin D and left to stand for 20 min at RT. Both supernatant and pellet were mixed or resuspended in equal amounts of SDS sample buffer. The samples were separated by SDS-PAGE and Western blot was performed as described below. The apparent Kd was obtained by non-linear fitting of the data using Prism (8.3.0) based on duplicate data allowing different values of the maximum P/S ratio for each group.

### 4.6. Western Blotting

Proteins were separated on 10% Sodium dodecyl sulfate polyacrylamide gel electrophoresis (SDS-PAGE) gels and transferred onto nitrocellulose membranes with semi-dry or wet transfer systems (BioRad, Hercules, CA, USA), depending on the size of proteins to be analyzed. The membranes were stained with Ponceau red protein stain for 15 min, rinsed with ddH_2_O and scanned with a CanoScan 9000F Mark II scanner (Canon). The Ponceau red was washed off and the membrane blocked with 5% milk in PBS-Tween20 for 1 h at RT or overnight at 4 °C. The antibodies were diluted in 5% milk PBS-Tween20 at 1:15,000 for anti-coronin A, and 1:5000 for anti-actin. Primary antibody incubation was done either at room temperature for 2 h or overnight at 4 °C, followed by washes and by incubation with horseradish peroxidase (HRP)-coupled secondary antibodies (Southern Biotech). Membranes were developed using SuperSignal PicoWest chemiluminescence substrate (Thermo-Fisher) or WesternBright Quantum HRP substrate (Advansta) and imaged using a Fuji FPM 800A (Fuji, Tokyo, Japan) or Fusion FX7 (VILBER, Paris, France)

### 4.7. Phagocytosis

For the preparation of particles, harvested log-growth phase *Klebsiela aerogenes* and *S. cerevisiae* (strain NYYO-1, [77]) were washed with and resuspended in KK2 buffer (16 mM KH_2_PO_4_, 4 mM K_2_HPO_4_) before heat killing at 80 °C and 65 °C, respectively, for 20 min. Heat-killed bacteria and yeast cells were stained in the dark, respectively, with 10× and 2.5× manufacturer recommended working concentration of CellBrite Fix 640 dye (#30089, Biotium, Freemont, CA, USA) for 30 min at RT. Excess dye solution was removed by centrifugation and labelled cell were resuspended in KK2 buffer. Live bacteria were laboratory strain *E. coli* (DH5α) expressing neon green fluorescent protein grown overnight in liquid broth to stationary growth phase. The live bacteria were a kind gift from Dirk Bumann at the Biozentrum, University of Basel, Basel, Switzerland. For beads, Ø = 1 µm, 3 µm, 4.5 µm or 6 µm fluorescent carboxylate-modified microspheres particles were obtained from Life technologies or PolySciences. For the uptake experiment, 12 × 10^7^ *Dictyostelium* cells were harvested in early log growth and resuspended in 6 mL HL5 medium at a density of 2 × 10^6^ cells/mL in a 2 conical flask and then incubate at 22 °C for 1 h at 160 rpm. For control, cells were pretreated with the actin depolymerizing drug cytochalasin A (C6637 Sigma-Aldrich) to a final concentration of 5 µg/mL for 30 min at 22 °C before the fluorescent particles were added. Particles were added to the *Dictyostelium* cells at MOI of 5, 10, 100, or 200 and incubated at 22 °C with 160 rpm, or at 4 °C (control) and 500 µL samples were collected at 0, 10, 20, 30, 60, and 90 min into 3 mL ice-cold KK2 supplemented with 5 µM NaN_3,_ washed and resuspended in 250 µL ice cold FACS buffer (PBS, 2% FCS, 10 mM EDTA, 0.05% Na-azide) and maintained on ice until measurement [78]. Non-ingested fluorescently labelled bacteria and yeast particles were quenched by adding 0.4% trypan blue at a ratio of 2:1 (trypan blue:sample) and incubated for 10 min prior to analysis. Samples were analyzed by Fluorescent Activated Cell Sorting (FACS) on a BD LSRFortessa (Becton-Dickinson, Franklin Lakes, NJ, USA) and FlowJo Software (flowjo.com).

## Supplementary Materials

Supplementary materials can be found at https://www.mdpi.com/1422-0067/21/4/1469/s1. Figure S1: Control for phagocytosis, Table S1: Table S1_Summary Sheet for all time points plus the combined, Table S2: Log2ratios_FLAG-CorA.
