# Supplementary material for: Interactome and F-Actin Interaction Analysis of Dictyostelium discoideum Coronin A
Source: Int J Mol Sci. 2020 Feb 21;21(4):1469. doi: 10.3390/ijms21041469 (PMC7073074; doi:10.3390/ijms21041469)
Supplement: Supplementary file 1 [file ijms-21-01469-s001.zip › Suppl data/Table S2_Log2ratios_FLAG-CorA.pdf]

Table S2

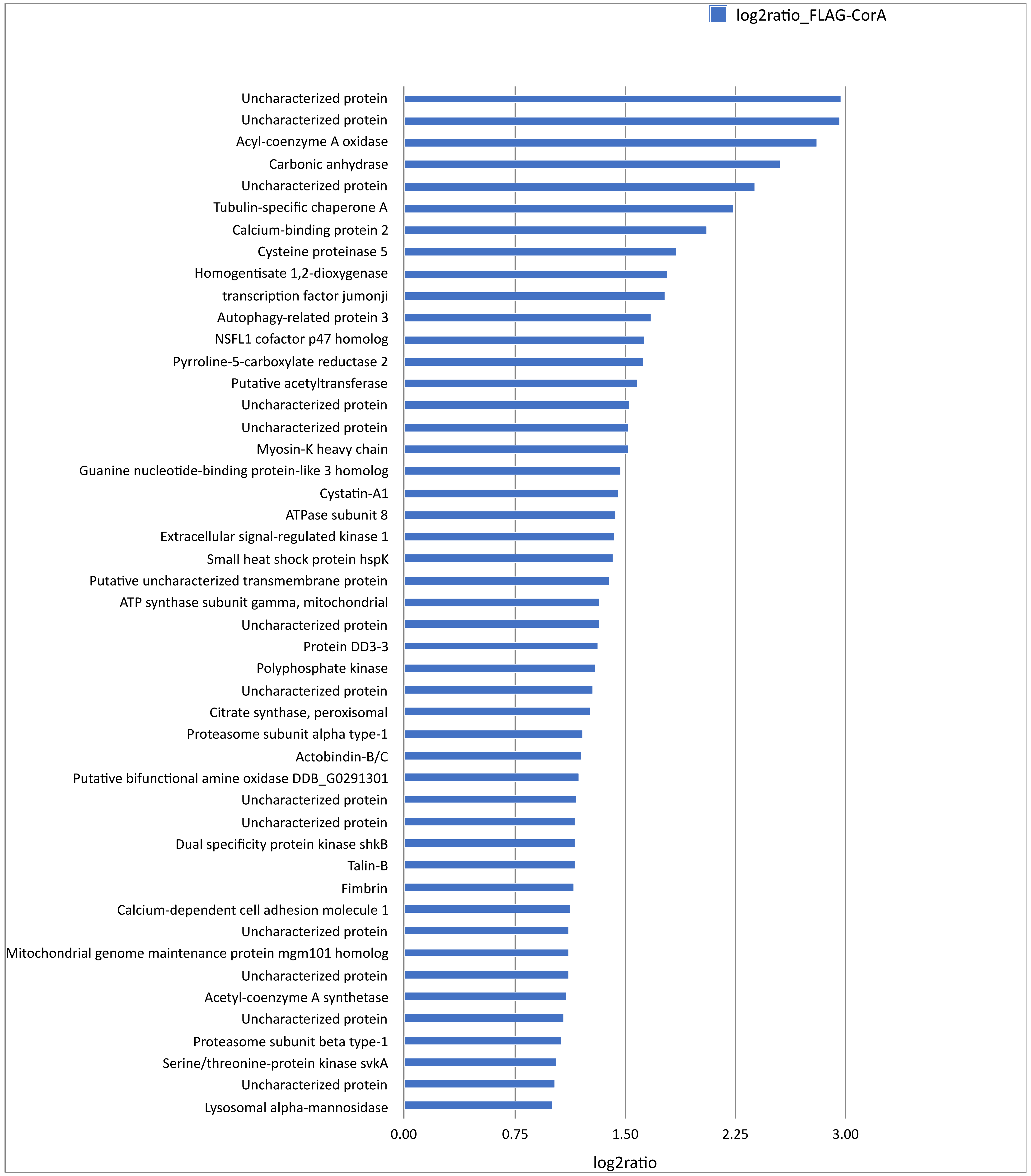

Accession Number	Gene Name	Protein Description	log2ratio_condition2_FLAG-CorA
Q54IG1	DDB0219382	Uncharacterized protein	2.97
Q58A40	DD7-1	Uncharacterized protein	2.96
Q54LF1	DDB_G0286669	Acyl-coenzyme A oxidase	2.80
Q55BU2	cahA	Carbonic anhydrase	2.55
Q86J14	DDB0167842	Uncharacterized protein	2.38
Q75JC8	tbca	Tubulin-specific chaperone A	2.24
P54653	cbp2	Calcium-binding protein 2	2.05
P54640	cprE	Cysteine proteinase 5	1.85
Q54QI4	hgd	Homogentisate 1,2-dioxygenase	1.79
Q54K96	jcdg	transcription factor jumonji	1.77
Q550A8	atg3	Autophagy-related protein 3	1.67
Q54BQ5	nsfl1c	NSFL1 cofactor p47 homolog	1.63
Q55E34	pycr2	Pyrroline-5-carboxylate reductase 2	1.63
Q54UU2	DDB_G0280825	Putative acetyltransferase	1.58
Q55DC0	DDB0190490	Uncharacterized protein	1.53
Q54FV6	DDB0188965	Uncharacterized protein	1.52
Q9XXV8	myoK	Myosin-K heavy chain	1.52
Q54KS4	gnl3	Guanine nucleotide-binding protein-like 3 homolog	1.47
Q65YR8	cpiA	Cystatin-A1	1.45
A9CLV7	atp8	ATPase subunit 8	1.43
P42525	erkA	Extracellular signal-regulated kinase 1	1.43
Q86H60	hspK	Small heat shock protein hspK	1.42
Q54UF5	DDB_G0281105	Putative uncharacterized transmembrane protein	1.39
Q54DF1	atp5C1	ATP synthase subunit gamma, mitochondrial	1.33
Q55GC0	DDB0189501	Uncharacterized protein	1.33
Q58A42	DD3-3	Protein DD3-3	1.31
Q54BM7	ppkA	Polyphosphate kinase	1.30
Q54HM2	DDB_G0289357	Uncharacterized protein	1.28
Q8MQU6	cshA	Citrate synthase, peroxisomal	1.27
Q27562	psmA1	Proteasome subunit alpha type-1	1.21
Q55DU1	abnB	Actobindin-B/C	1.21
Q54EW2	DDB_G0291301	Putative bifunctional amine oxidase DDB_G0291301	1.19
Q54V27	lmcA	Uncharacterized protein	1.17
Q54G77	DDB0188843	Uncharacterized protein	1.16
Q54IP4	shkB	Dual specificity protein kinase shkB	1.16
Q54K81	talB	Talin-B	1.16
P54680	fimA	Fimbrin	1.15
P54657	cadA	Calcium-dependent cell adhesion molecule 1	1.13
Q55GG3	DDB_G0268498	Uncharacterized protein	1.12
Q8MYF0	mgm101	Mitochondrial genome maintenance protein mgm101 homolog	1.12
Q86K89	DDB0169073	Uncharacterized protein	1.12
Q54Z60	acsA	Acetyl-coenzyme A synthetase	1.10
Q54Q32	DDB0185860	Uncharacterized protein	1.09
Q86A21	psmB1	Proteasome subunit beta type-1	1.07
O61122	svkA	Serine/threonine-protein kinase svkA	1.03
Q54PJ6	DDB_G0284509	Uncharacterized protein	1.02
P34098	manA	Lysosomal alpha-mannosidase	1.00
