# Supplementary material for: Interactome and F-Actin Interaction Analysis of Dictyostelium discoideum Coronin A
Source: Int J Mol Sci. 2020 Feb 21;21(4):1469. doi: 10.3390/ijms21041469 (PMC7073074; doi:10.3390/ijms21041469)
Supplement: Supplementary file 1 [file ijms-21-01469-s001.zip › Suppl data/Figure S1_phagocytosis controls.pdf]

Figure S1

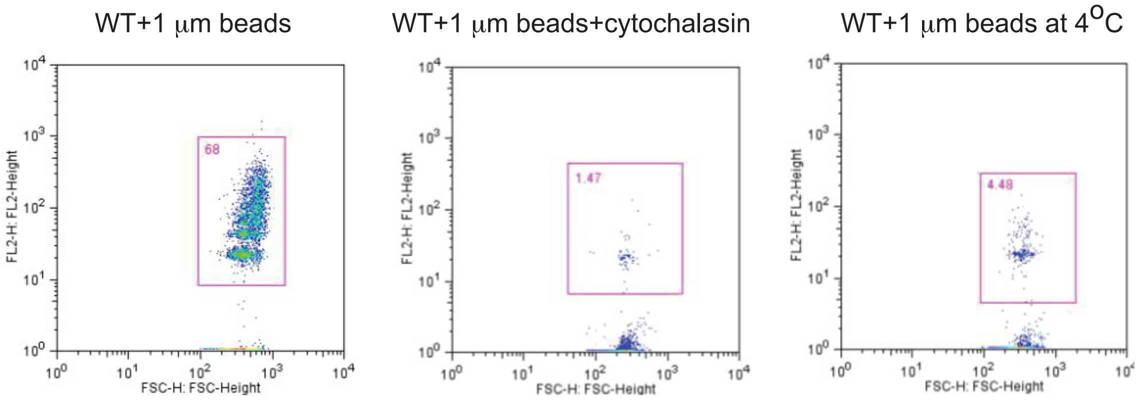

Figure S1: Control experiments for phagocytosis
Representative example of 68 % of the DH1.10 wild-type cells containing at least 1 bead (1 µm) while treatment with cytochalasin or 4oC incubation showed less than 5% of the cells containing at least 1 latex bead. Phagocytosis of the particles in general was strongly inhibited to less than 15% of cells containing particles for both controls for all other conditions.
